# A blend of selected botanicals maintains intestinal epithelial integrity and reduces susceptibility to *Escherichia coli* F4 infection by modulating acute and chronic inflammation *in vitro*

**DOI:** 10.3389/fvets.2023.1275802

**Published:** 2023-09-29

**Authors:** Andrea Bonetti, Andrea Toschi, Benedetta Tugnoli, Andrea Piva, Ester Grilli

**Affiliations:** ^1^Dipartimento di Scienze Mediche Veterinarie (DIMEVET), Università di Bologna, Ozzano dell’Emilia, Bologna, Italy; ^2^Vetagro S.p.A., Reggio Emilia, Italy; ^3^Vetagro Inc., Chicago, IL, United States

**Keywords:** intestinal health, inflammation, oxidative stress, enterotoxigenic *Escherichia coli*, botanicals

## Abstract

In the pig production cycle, the most delicate phase is weaning, a sudden and early change that requires a quick adaptation, at the cost of developing inflammation and oxidation, especially at the intestinal level. In this period, pathogens like enterotoxigenic *Escherichia coli* (ETEC) contribute to the establishment of diarrhea, with long-lasting detrimental effects. Botanicals and their single bioactive components represent sustainable well-recognized tools in animal nutrition thanks to their wide-ranging beneficial functions. The aim of this study was to investigate the *in vitro* mechanism of action of a blend of botanicals (BOT), composed of thymol, grapeseed extract, and capsicum oleoresin, in supporting intestinal cell health during inflammatory challenges and ETEC infections. To reach this, we performed inflammatory and ETEC challenges on Caco-2 cells treated with BOT, measuring epithelial integrity, cellular oxidative stress, bacterial translocation and adhesion, gene expression levels, and examining tight junction distribution. BOT protected enterocytes against acute inflammation: while the challenge reduced epithelial tightness by 40%, BOT significantly limited its drop to 30%, also allowing faster recovery rates. In the case of chronic inflammation, BOT systematically improved by an average of 25% the integrity of challenged cells (*p* < 0.05). Moreover, when cells were infected with ETEC, BOT maintained epithelial integrity at the same level as an effective antibiotic and significantly reduced bacterial translocation by 1 log average. The mode of action of BOT was strictly related to the modulation of the inflammatory response, protecting tight junctions’ expression and structure. In addition, BOT influenced ETEC adhesion to intestinal cells (−4%, *p* < 0.05), also thanks to the reduction of enterocytes’ susceptibility to pathogens. Finally, BOT effectively scavenged reactive oxygen species generated by inflammatory and H_2_O_2_ challenges, thus alleviating oxidative stress by 40% compared to challenge (*p* < 0.05). These results support the employment of BOT in piglets at weaning to help manage bacterial infections and relieve transient or prolonged stressful states thanks to the modulation of host-pathogen interaction and the fine-tuning activity on the inflammatory tone.

## 1. Introduction

To cope with worldwide increasing meat demands (1), large-scale animal production systems have adopted intensive management procedures that expose animals to a wide variety of stressful stimuli that deeply impair their health and performance. When animals experience these pressures, they lose their homeostatic state to undergo an adaptation phase: if the new environmental conditions require considerable changes, stress is developed (2). This reaction is usually unfavorable to farmers, who need high animal resilience to ensure maximum production at the lowest cost.

For pigs, weaning represents the most delicate moment of their life, during which they experience sudden and dramatic changes in a short period of time. Common practice suggests that weaning should be carried out around 21–28 days of age, when piglets have not completed their gastrointestinal maturation (3), with consequences that can last for the entire production cycle (4, 5). The gastrointestinal tract of piglets is particularly affected by early weaning practices: the loss of intestinal integrity, the reduction of digestive enzyme production, the decrease in the absorptive surface, the arrival of new dietary antigens, and the lack of a full immune competence result in a state of considerable intestinal stress (6–8).

This detrimental condition triggers the onset of a prolonged inflammatory state, which impairs intestinal homeostasis and worsens the oxidative status of the gut mucosa (9, 10). Bacteria can take advantage of the host’s susceptibility to overgrow in the intestinal lumen: the most frequent infection in weaning piglets is caused by enterotoxigenic *Escherichia coli* (ETEC), a group of pathogens that exacerbates the onset of diarrhea, further damaging the overall health and performance of piglets (11, 12).

Weaning stress and its symptoms, like diarrhea, were traditionally treated with antibiotics and pharmacological doses of zinc oxide (12, 13). The employment of antibiotics is now mainly restricted to full-blown cases, under veterinary prescription, and seriously limited due to the ever-expanding phenomenon of antimicrobial resistance, which is endangering the efficacy of antibiotics for both human and animal medicine (12, 14, 15). Similarly, high zinc oxide doses were banned in several areas of the world, such as the European Union, because of environmental pollution and bacterial resistance concerns (16).

Ground-breaking sustainable alternatives are urgently required to handle animal stress. A wide and varied source of novel and diverse compounds is represented by nature since plants innately produce numerous molecules to defend themselves against pathogens, predators, and stressors (17, 18). Botanicals such as essential oils, oleoresins, and powder extracts are broadly studied in animal nutrition thanks to their recognized antimicrobial, antioxidant, and anti-inflammatory properties (19, 20). Our previous studies showed that certain single terpene-rich botanicals and phenol-rich botanicals were able to modulate oxidative stress, ameliorate epithelial integrity of enterocytes, and support cells to manage ETEC infections *in vitro*, with specific mechanisms of action and peculiar individual properties (21, 22).

In this framework, the aim of this study was to investigate the ability of a blend of selected botanicals (BOT) in different stressful conditions on cultured Caco-2 enterocytes, a well-known model for intestinal studies. In particular, we assessed BOT ability to modulate acute and chronic inflammatory challenges, and explored its efficacy against an ETEC infection, while also elucidating its in-depth mechanism of action *in vitro*.

## 2. Materials and methods

### 2.1. Chemicals and reagents

Chemicals and cell culture reagents were provided by Merck KGaA (Darmstadt, Germany) unless otherwise specified. Capsicum oleoresin was purchased from Frey&Lau (Frey + Lau GmbH, Henstedt-Ulzburg, Germany) and grape seed extract was obtained from Layn Natural Ingredients (Guilin Layn Natural Ingredients Corp., Shanghai, China). The blend of botanicals (BOT) was composed of thymol, grape seed extract, and capsicum oleoresin, and tested at a concentration of 30 ppm (10 ppm of each component) in all the experiments. Stock solutions of the BOT components were prepared in ethanol 100% (v/v) and supplemented in a culture medium at a final working concentration of ethanol ≤1% (v/v).

### 2.2. Cell line and culture conditions

The human colon adenocarcinoma cell line (Caco-2) was obtained from DSMZ (DSMZ-German Collection of Microorganisms and Cell Cultures, Leibniz Institute, Germany). Caco-2 cells were maintained in a basal medium composed of Dulbecco’s modified Eagle’s medium (DMEM) supplemented with 10% fetal bovine serum (FBS), 1% L-glutamine, 1% non-essential amino acids, and 1% penicillin/streptomycin (P/S). Cells were incubated at 37°C in an atmosphere containing 5% CO_2_ at 95% relative humidity.

### 2.3. Inflammatory challenge

To evaluate the monolayer integrity, Caco-2 cells were cultured into 24-well Transwell^®^ inserts (0.4 μm diameter pores) (Corning, Massachusetts, United States), seeded at a density of 5 × 10^4^ cells/transwell. Transepithelial Electrical Resistance (TER) was measured using an epithelial tissue voltohmmeter (Millicell ERS-2, Merk, Darmstadt, Germany). The experiment started 28 days after the seeding on filters when Caco-2 cells reached a TER value >600 Ω cm^2^ which indicates adequate monolayer integrity and differentiation.

#### 2.3.1. Acute inflammatory challenge

Once completely differentiated, Caco-2 cells were divided into five groups: a negative control group (CTR-) maintained in basal medium for the entire duration of the experiment, two positive control groups (CTR+) that were maintained in basal medium and challenged either at day 0 or day 6, and two treated groups (BOT+) that were supplemented with the blend of botanicals and challenged either at day 0 or day 6.

The acute inflammatory challenge was performed as described by Toschi et al., (23). Briefly, challenged cells were exposed for 24 h to a cocktail of pro-inflammatory cytokines (cytomix) including IL-1β (25 ng/mL), TNFα (50 ng/mL), and IFNγ (50 ng/mL), and LPS from *E. coli* O55:B5 (10 μg/mL). Depending on the experimental group, challenged cells received the cytomix + LPS either at day 0 (early acute inflammatory challenge) or at day 6 (late acute inflammatory challenge). Throughout the experiment, TER was measured on days 0, 1, 2, 5, 6, and 7.

#### 2.3.2. Chronic inflammatory challenge

The chronic inflammatory challenge was adapted from Toschi et al., (23). To obtain continuous inflammatory stimulation on cells, the challenge was performed for 7 days with basal medium containing cytomix + LPS. The inflammatory cocktail was refreshed every 2 days adding incremental doses of LPS (10, 20, and 30 μg/mL on days 2, 4, and 6, respectively) to avoid eventual adaptation.

Cells were divided into 4 groups depending on the presence of the challenge and BOT (CTR-, CTR+, BOT-, BOT+), and treated for a total of 7 days during which TER was measured at days 0, 1, 2, 3, 6, and 7. At the end of the experiment, cells were washed with DPBS and harvested for gene expression analysis.

#### 2.3.3. Measurement of reactive oxygen species (ROS) levels

ROS were measured using CellROX^®^ Deep Red Reagent (Thermofisher Scientific, Milan, Italy) following the manufacturer’s instructions. To perform the analysis, Caco-2 cells were seeded at a density of 1.5 × 10^4^ cells/well onto 96-well plates and maintained in basal medium. Once reached the confluence, cells were treated with BOT for 24 h. Then, challenged groups were supplemented with cytomix + LPS for 24 h or 500 μM H_2_O_2_ for 1 h to induce ROS production before CellROX^®^ assay. Fluorescence values were recorded with Varioskan^™^ LUX (Thermofisher Scientific, Waltham, MA, United States).

### 2.4. Bacterial challenge

The pathogen employed for the bacterial challenge was a field strain of ETEC with F4 adhesins, expressing heat-labile and heat-stable toxins (LT^+^, STa^+^, STb^+^), and originally isolated from a weaning piglet affected by post-weaning diarrhea. Bacteria were cultured in brain-heart infusion broth (BHI, VWR International, Milan, Italy) at +37°C and daily passaged 1:100 to maintain an active culture (24). Caco-2 cells were obtained and routinary maintained as described in Section 2.2.

On challenge day, the bacterial inoculum was prepared by passing 1:80 the overnight culture in fresh BHI. After 4 h, when the late logarithmic growth phase was reached, bacterial turbidity at 630 nm was measured at the spectrophotometer. The resulting value was interpolated into an absorbance – CFU/mL curve previously prepared in order to standardize the bacterial inoculum at a precise concentration for the infection experiments, as previously described by Roselli et al. (25).

#### 2.4.1. Infection of Caco-2 cells on porous filters

To recreate an ETEC infection on intestinal cells *in vitro*, Caco-2 cells were differentiated on porous Transwell^®^ inserts (3.0 μm diameter pores) (Corning, Massachusetts, United States) in 12 well plates for 30 days.

The infection protocol was performed according to our previous study (22). Briefly, Caco-2 were differentiated and TER of all inserts measured as described in paragraph 2.3. Then, cells were washed twice with DPBS, and infected on the apical side with 5 × 10^7^ CFU/mL ETEC (multiplicity of infection of 100) in basal medium without P/S and supplemented with the blend of botanicals (BOT+). Also the basolateral side of inserts contained the BOT treatment in basal medium without P/S. The experiment included three controls: a negative control (CTR-) without bacteria, a positive control (CTR+) with only the bacteria, and another group with the bacteria and 4 ppm colistin, a dose able to completely kill bacteria (COL+).

TER was measured at 2 and 4 h after the beginning of the infection to assess cellular integrity. At both time points, 100 μL of basolateral medium was collected from each filter and immediately diluted into sterile saline. Aliquots of the most appropriate dilutions were seeded on BHI agar plates, incubated for 24 h at +37°C, and then counted to enumerate viable bacteria translocated across the Caco-2 monolayer (bacterial translocation, BT).

To allow gene expression analysis, at the end of the experiment cells were washed twice with DPBS, then harvested and stored at −80°C until RNA extraction.

#### 2.4.2. Adhesion assay

As previously described (22), to assess the ability of BOT to influence bacterial interaction with target cells, Caco-2 were differentiated on 24 well plates. On the infection day, cells were washed twice with DPBS, then infected with 5 × 10^7^ CFU/mL ETEC in basal medium without P/S and supplemented with the blend of botanicals (BOT+). The experimental setup also included two controls: a positive control (CTR+) with only bacteria, and a group supplemented with bacteria and 4 ppm colistin (COL+), a dose able to completely kill bacteria.

After a 1 h infection, non-adhered bacteria were discarded, and then cells were washed four times with DPBS. Caco-2 was lysed with 0.5% Triton X-100 in DPBS for 10 min, then serially diluted in sterile saline to plate the most appropriate dilutions on BHI agar. Seeded plates were incubated for 24 h at +37°C, and viable bacteria adhered to cells were finally counted.

### 2.5. Gene expression assay

Gene expression was performed on cells harvested at the end of the chronic inflammatory challenge and the bacterial challenge. The assay’s protocol was based on previous studies (21, 23). Briefly, RNA was extracted using NucleoSpin RNA Kit (Macherey-Nagel, Düren, Germany) with DNase digestion according to manufacturer’s instructions. The RNA yield and purity were assessed by A230, A260, and A280 nm measurements at the spectrophotometer (μDrop Plate and Varioskan LUX, Thermo Fisher Scientific, Waltham, MA, United States).

RNA was reverse-transcribed using iScript cDNA Synthesis Kit (Bio-Rad Laboratories, Hercules, CA, United States) according to the manufacturer’s instructions. Then, cDNA was diluted and used for qPCR in reaction mixes prepared with iTaq Universal SYBR Green Supermix (Bio-Rad Laboratories, Hercules, CA, United States). Table 1 shows forward and reverse primer sequences obtained from Merck (Darmstadt, Germany) and used for all the selected target genes.

**Table 1:** Primers used for gene expression experiments in this study.

Function	Gene	Sequences (5′ ➔ 3′)	Product length (bp)	Ref.
Tight-junction integrity	*ZO-1*	F: CGGGACTGTTGGTATTGGCTAGA R: GGCCAGGGCCATAGTAAAGTTTG	184	(26)
	*ZO-2*	F: CTAGCAGCGATCAACTTAGGGACAA R: CCCAGGAGTTTCATTACCAGCAA	158	(26)
	*CLD-1*	F: GCACATACCTTCATGTGGCTCAGF R: TGGAACAGAGCACAAACATGTCA	92	(26)
	*OCCL*	F: TCCTATAAATCCACGCCGGTTC R: CTCAAAGTTACCACCGCTGCTG	105	(26)
Innate immune response	*TNFα*	F: TCTCGAACCCCGAGTGACAA R: TATCTCTCAGCTCCACGCCA	124	(27)
	*IL-1β*	F: AATCTGTACCTGTCCTGCGTGTT R: TGGGTAATTTTTGGGATCTACACTCT	78	(28)
	*IL-6*	F: AGCCCTGAGAAAGGAGACATGT R: AGGCAAGTCTCCTCATTGAATCC	141	(28)
	*IL-8*	F: ATGACTTCCAAGCTGGC R: ACTTCTCCACAACCCT	174	(29)
Cellular response to ETEC	*MUC13*	F: CGCTTGTCAGAGAGGTGGTT R: AATGCTGGGGAGCTTTCCTC	131	This study
	*BD1*	F: CCTACCTTCTGCTGTTTACTC R: ACTTGGCCTTCCCTCTGTAAC	186	(30)
	*GUCY2C*	F: CGGGTGGCTGTCCTTTAGTT R: AGGCTGAGTTGCCCATCATC	89	This study
Housekeeping genes	*RPLP0*	F: GCAATGTTGCCAGTGTCTG R: GCCTTGACCTTTTCAGCAA	142	(31)
	*GAPDH*	F: TGCACCACCAACTGCTTAGC R: GGCATGGACTGTGGTCATGAG	87	(32)

Ref, reference; F, forward; R, reverse; ZO-1, zonula occludens 1; ZO-2, zonula occludens 2; CLD-1, claudin 1; OCCL, occludin; TNFα, tumor necrosis factor α; IL-1β, interleukin 1β; IL-6, interleukin 6; IL-8, interleukin 8; MUC13, mucin 13; BD1, beta defensin 1; GUCY2C, guanylyl cyclase 2C; RPLP0, ribosomal protein lateral stalk subunit P0; GAPDH, glyceraldehyde 3 phosphate dehydrogenase.

qPCR analysis was performed by using a CFX96 Real-Time PCR Detection System (Bio-Rad Laboratories, Hercules, CA, United States) under the following conditions: 3 min at 95°C, followed by 40 cycles of 95°C for 10 s and 60°C for 30 s. The specificity of each reaction was evaluated by melting-curve analysis.

After collecting threshold cycles, gene expression levels were normalized using two reference genes, i.e., ribosomal protein lateral stalk subunit P0 (RPLP0) and glyceraldehyde-3-phosphate dehydrogenase (GAPDH). Relative changes in gene expression were calculated using the 2^−ΔΔCt^ method (33).

### 2.6. Immunofluorescence staining

To investigate the morphology of cells that experienced an inflammatory challenge or a bacterial infection, Caco-2 cells were stained by an immunofluorescence assay for zonula occludens 1 (ZO-1), a tight junction protein.

After having differentiated Caco-2 on glass coverslips (10 mm diameter) placed at the bottom of 6 well plates, with each well corresponding to a different group of treatment, cells were washed twice with DPBS, then challenged 24 h with cytomix + LPS or 2 h with 5 × 10^7^ CFU/mL ETEC in basal medium without P/S and supplemented with the blend of botanicals (BOT+). The experimental design also included two controls: a negative control (CTR-) without any treatment or challenge, and a positive control (CTR+) with only the cytomix + LPS or bacteria.

After the challenge, cells were washed twice with DPBS, and then fixed in 4% paraformaldehyde in DPBS. Staining was performed according to our previous study (22). Briefly, Caco-2 were permeabilized with 0.5% Triton X-100, and subsequently blocked with 10% goat serum. Then, the rabbit anti-ZO-1 primary monoclonal antibody (ThermoFisher Scientific, Walthan, MA, United States) was diluted following manufacturer’s instructions in DPBS with 2% bovine serum albumin (BSA) and 0.05% saponins and incubated on cells for 3 h at +4°C in humidified atmosphere. After three washes, a goat anti-rabbit secondary antibody conjugated to fluorescein isothiocyanate (FITC) (ThermoFisher Scientific, Walthan, MA, United States) was employed to bind the primary antibody for 1 h. After washing, nuclei were counterstained and slides mounted with Fluoroshield containing 4′,6-diamidino-2-phenylindole (DAPI). Pictures were visualized and acquired using a fluorescent microscope and images elaborated with NIS-Elements software (Nikon Corporation, Tokyo, Japan).

### 2.7. Statistical analysis

For each experiment, the experimental unit was the well, with *n* = 6 for each treatment group. Data are displayed on graphs as means ± SEM. All data were processed using GraphPad Prism v.9.5.0 (GraphPad Software, Inc., San Diego, CA, United States). TER and BT were analyzed with Two-Way ANOVA analysis with Tukey post-hoc test comparing all experimental groups with each other. Adhesion assay, gene expression, and oxidative stress data were evaluated with One-Way ANOVA analysis with Tukey post-hoc test, comparing all experimental groups with each other. Differences were considered significant when *p* ≤ 0.05, and trends were identified when 0.05 < *p* ≤ 0.1.

## 3. Results

### 3.1. Inflammatory challenge

#### 3.1.1. Acute inflammatory challenge

**Figure 1:**
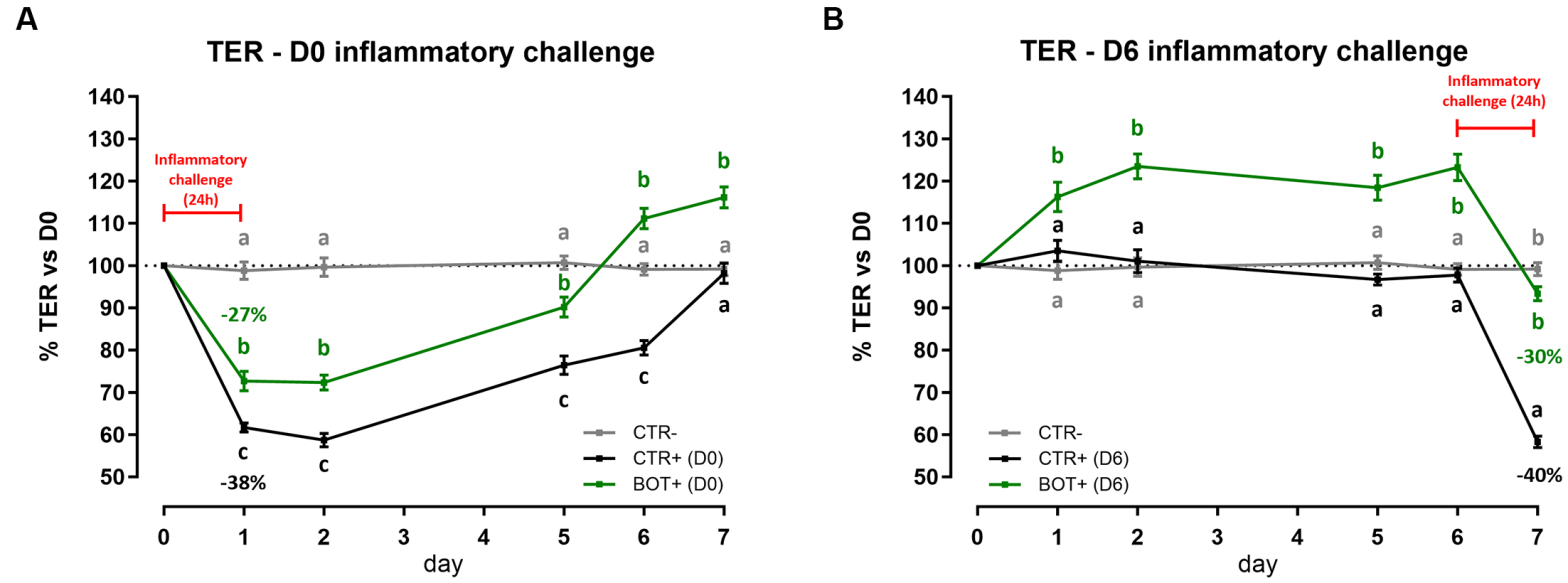
TER results during an acute LPS + cytomix challenge (24 h) on Caco-2 cells treated with a blend of botanicals (BOT). Panel **(A)** displays the TER of the challenge at D0, applied simultaneously to the treatment, while panel **(B)** shows the TER of the challenge at D6, applied on the last day of BOT treatment. Negative control (CTR-) was shared between the two challenges. Experimental groups with a “+” in the name were challenged. Data in graphs are represented as means ± SEM. Different letters indicate significant differences with *p* < 0.05 at each time point.

Figure 1 reports the results of the acute inflammatory challenge, performed either at D0 or at D6 of BOT treatment.

When the inflammatory challenge was performed at the beginning of the experiment (Figure 1A), data showed that BOT was able to limit the drop (−27%) of cellular integrity caused by the challenge itself (−38%). The moderate drop in TER was also followed by a faster and higher recovery during the subsequent days: the BOT group showed always TER values higher than positive control and reached TER levels in line with or greater than the starting basal value (100%) one day in advance.

Similar results were obtained when Caco-2 was pre-treated with BOT and challenged for 24 h at D6 (Figure 1B). The application of BOT significantly improved the basal TER of intestinal epithelial cells, with an average of +20% in the first 6 days compared to the control. In addition, BOT allowed the maintenance of a higher integrity after the application of the cytomix + LPS: the challenge reduced by 30% the TER of the BOT+ group, while the drop was higher for CTR+ (40%).

#### 3.1.2. Chronic inflammatory challenge

**Figure 2:**
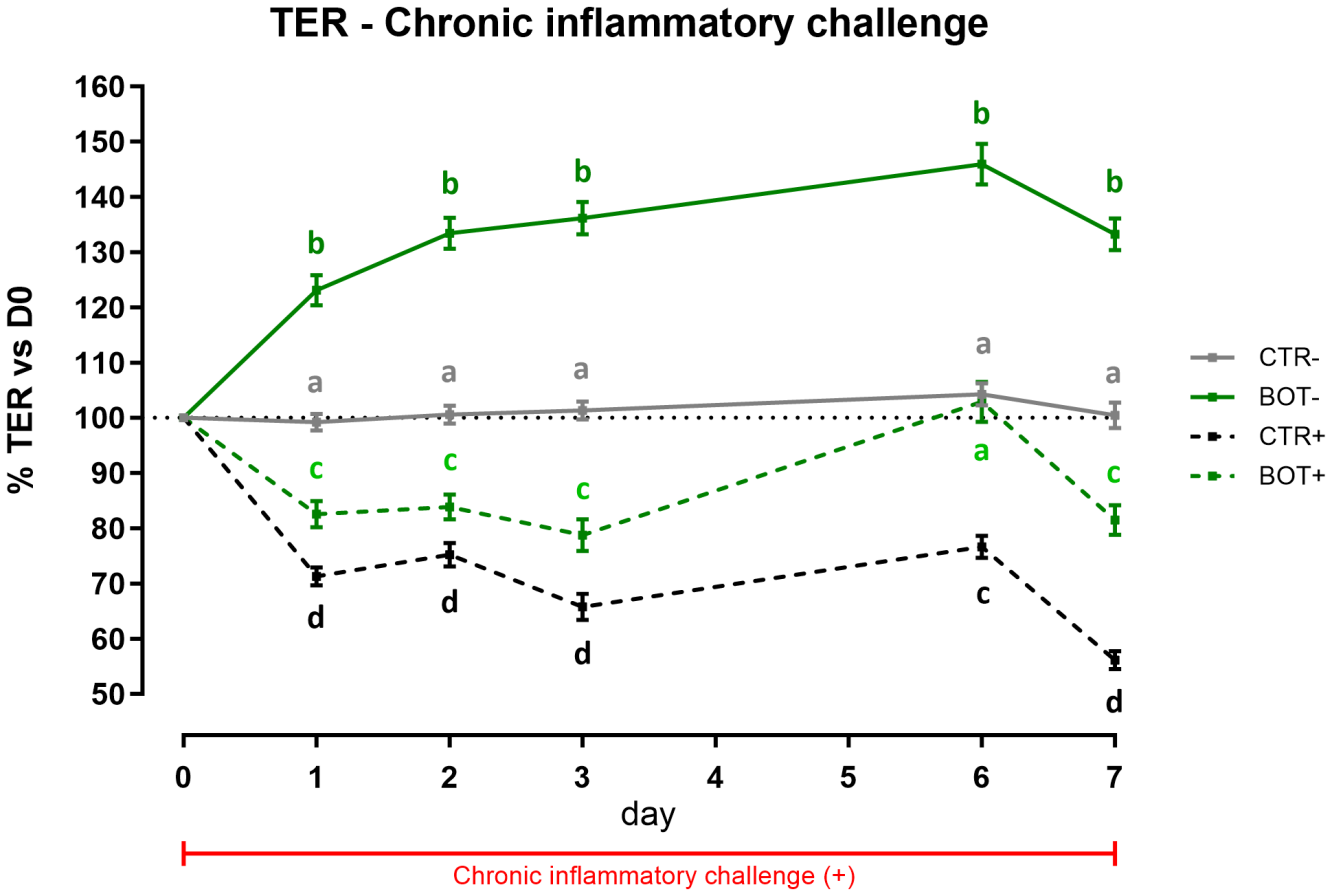
TER results during a chronic LPS + cytomix challenge (7 days) on Caco-2 cells treated with a blend of botanicals (BOT) for 7 days. Experimental groups with a “+” in the name were challenged. Data in graphs are represented as means ± SEM. Different letters indicate significant differences with *p* < 0.05 at each time point.

To investigate the effect of BOT on a prolonged inflammatory state on intestinal cells, Caco-2 was challenged for 7 days while simultaneously being treated with the blend of botanicals. Figure 2 displays the TER levels across the experiment. In normal conditions without the challenge, the treatment significantly increased the integrity of cells during the 7 days of treatment (BOT-) compared to control (CTR-), allowing enterocytes to reach a TER value up to 146% of the initial value on day 6.

The chronic inflammatory challenge (CTR+) systematically reduced cellular integrity during the experiment, keeping TER at values significantly lower than CTR-, between 75 to 55% of the initial ones. However, the BOT application consistently improved the integrity values of the BOT+ group, maintaining TER at levels significantly higher than the CTR+ group. On day 6, BOT+ integrity registered an enhancement that reached the unchallenged control (CTR-).

#### 3.1.3. ROS levels

**Figure 3:**
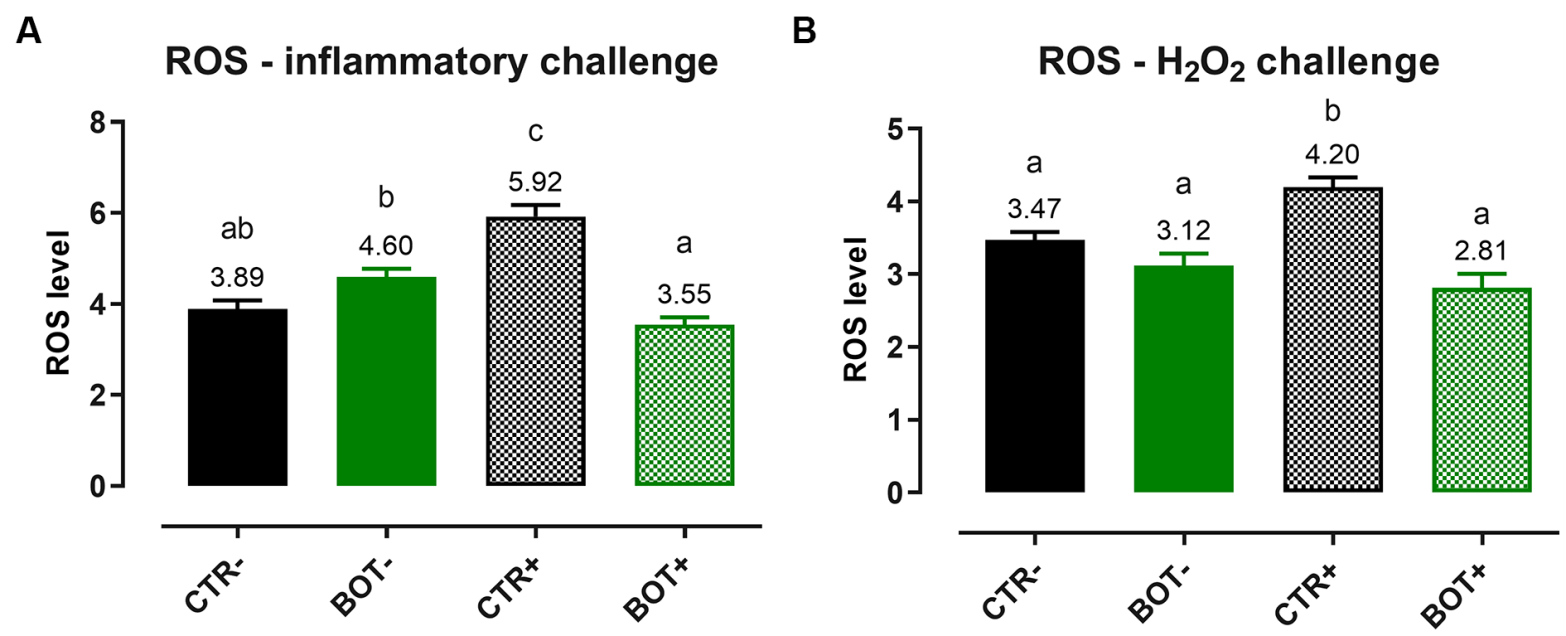
Reactive oxygen species levels in Caco-2 cells treated with a blend of botanicals (BOT) and challenged with LPS + cytomix for 24 h **(A)** or with hydrogen peroxide (H_2_O_2_) for 1 h **(B)**. Groups with the challenge are represented with a “+” in the name. Data in the graphs are reported as means ± SEM. Different letters denote significant differences with *p* < 0.05.

To examine the detailed mechanism of action of BOT, ROS levels were measured after both an inflammatory and an H_2_O_2_ challenge on Caco-2, and the results are reported in Figure 3. In both cases, when applied to normal intestinal cells, BOT did not significantly modify the ROS levels of the cellular system. However, in the presence of inflammatory and H_2_O_2_ challenges, both recognized to generate ROS, the application of BOT significantly reduced their production (−40% and −38%, respectively), restoring their concentration at the same level of the negative control (CTR-).

#### 3.1.4. Gene expression

Caco-2 cells were harvested and mRNA levels were assessed to further elucidate the action of BOT in maintaining cellular integrity and modulating the inflammatory response during the chronic challenge.

**Figure 4:**
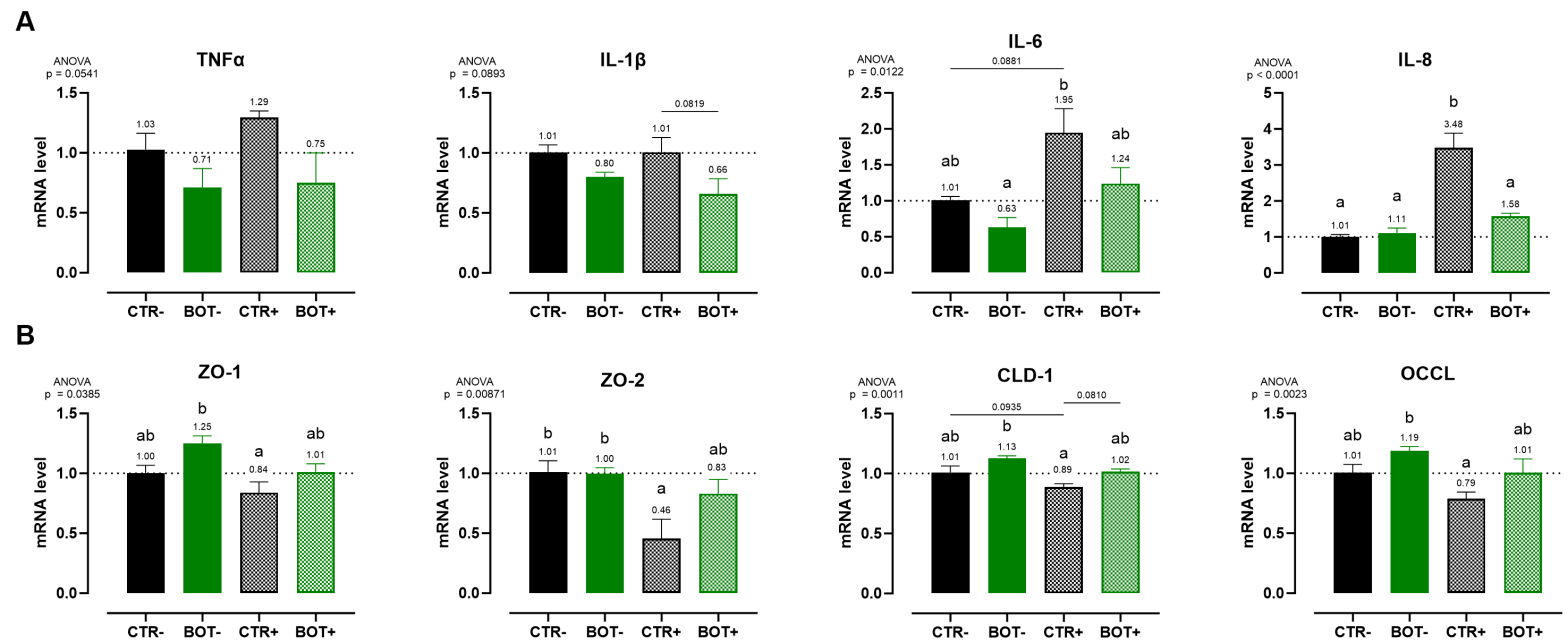
Gene expression analysis of Caco-2 cells treated with a blend of botanicals (BOT) and challenged with LPS + cytomix for 7 days. Panel **(A)** displays the inflammatory cytokines (TNFα, IL-1β, IL-6, IL-8), while panel **(B)** reports the data for tight-junction markers (ZO-1, ZO-2, CLD-1, OCCL). Groups with the inflammatory challenge are represented with a “+” in the name. Data in the graphs are reported as means ± SEM. Different letters denote significant differences with *p* < 0.05; tendencies (*p* < 0.1) are highlighted by reporting their respective *p* values and indicated by a line spanning the two evaluated groups.

Figure 4A shows the investigation of inflammation-related genes, like TNFα, IL-1β, IL-6, and IL-8. The challenge was able to increase the gene expression of IL-8 (*p* < 0.05) and IL-6 (*p* < 0.1), while for TNFα only numerical increases were measured, and no differences were registered for IL-1β. The BOT treatment alone, on healthy cells, did not significantly modify cytokines expression, even if some numerical reductions are displayed for TNFα, IL-1β, and IL-6. However, on challenged cells BOT was able to decrease the levels of IL-8 (*p* < 0.05) and IL-1β (*p* < 0.1) cytokines, with a numerical reduction for TNFα, and bringing back IL-6 expression in line with negative controls.

Figure 4B displays the expression levels of genes directly related to tight-junction integrity like zonula occludens 1 (ZO-1), zonula occludens 2 (ZO-2), claudin 1 (CLD-1), and occludin (OCCL). On healthy Caco-2 cells, BOT numerically increased the levels of ZO-1, CLD-1, and OCCL, with gene expression always significantly different from the positive control (CTR+). The inflammatory challenge (CTR+) worsened the expression of all the studied tight-junctions, with a significant effect on ZO-2, if compared to CTR-. The addition of BOT to the challenge enhanced the mRNA levels of all the studied tight-junction genes: although not significantly different from CTR+, gene expression was restored at the same level of the negative controls (CTR- and BOT-).

#### 3.1.5. Immunofluorescence staining

**Figure 5:**
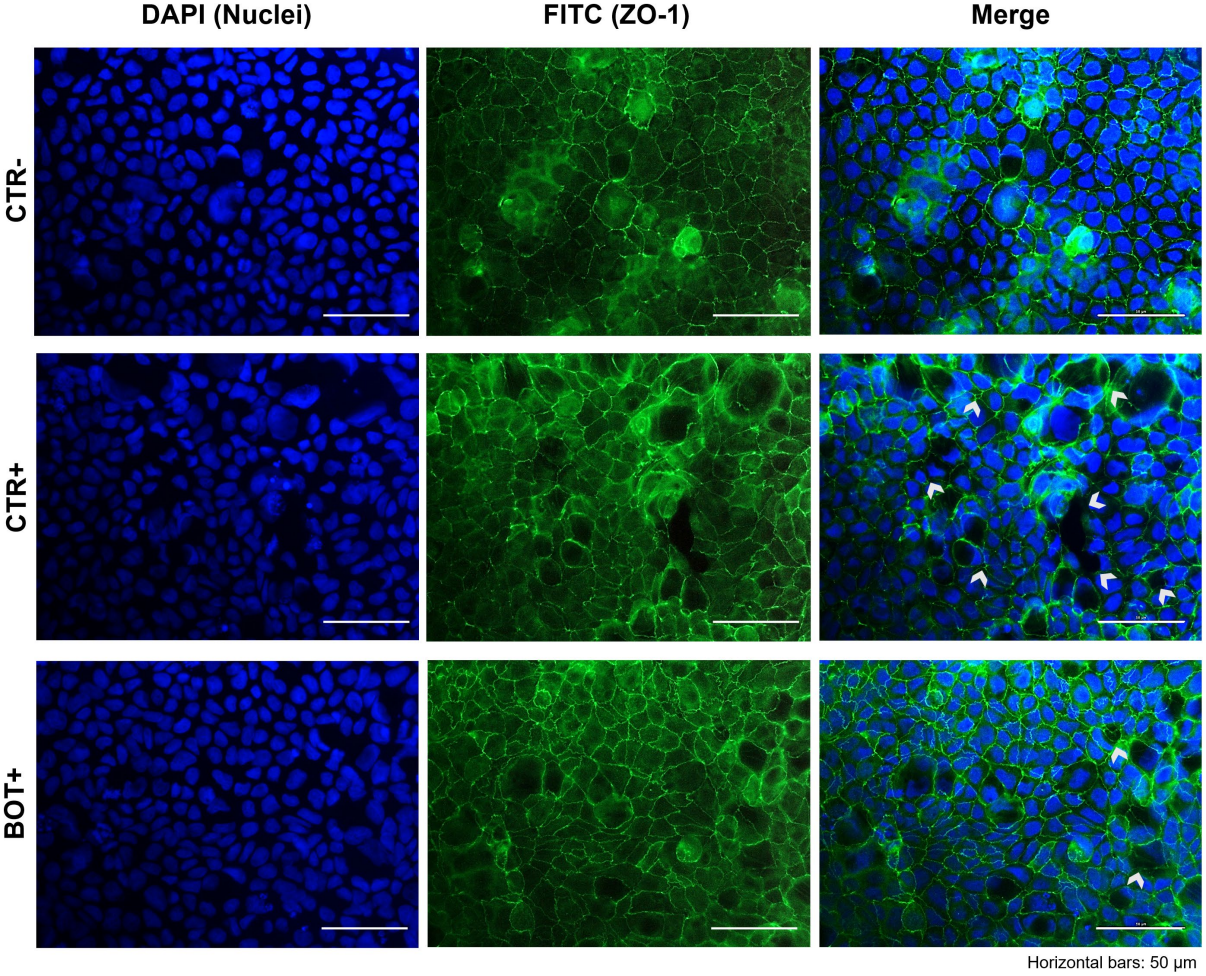
Immunofluorescence staining of Caco-2 cells treated with a blend of botanicals (BOT) and challenged with LPS + cytomix for 24 h. Groups with the inflammatory challenge are represented with a “+” in the name. The left pictures show the DAPI staining of cell nuclei, the central column depicts ZO-1 staining with FITC, while the right column displays the merge of the first two images. White arrows identify areas with anomalies of ZO-1 disposition, or loss of cells. Each row displays a different experimental group.

The immunofluorescence staining for ZO-1 of cells experiencing an inflammatory challenge with or without BOT is shown in Figure 5. The application of the cytomix + LPS produced apparent alterations to the morphology of Caco-2, which tended to organize without a regular and ordered pattern. This resulted in areas of irregular ZO-1 disposition, partial loss of contact between adjacent cells, and the presence of localized acellular zones as a result of the detachment of cells. The supplementation of BOT significantly helped enterocytes to cope with the negative effects of the challenge by reducing the observed damages, with only some minor sites of ZO-1 misplacement or loss of tightness. No localized acellular areas were detected.

### 3.2. Bacterial challenge

#### 3.2.1. Cellular integrity, bacterial translocation, and adhesion results

**Figure 6:**
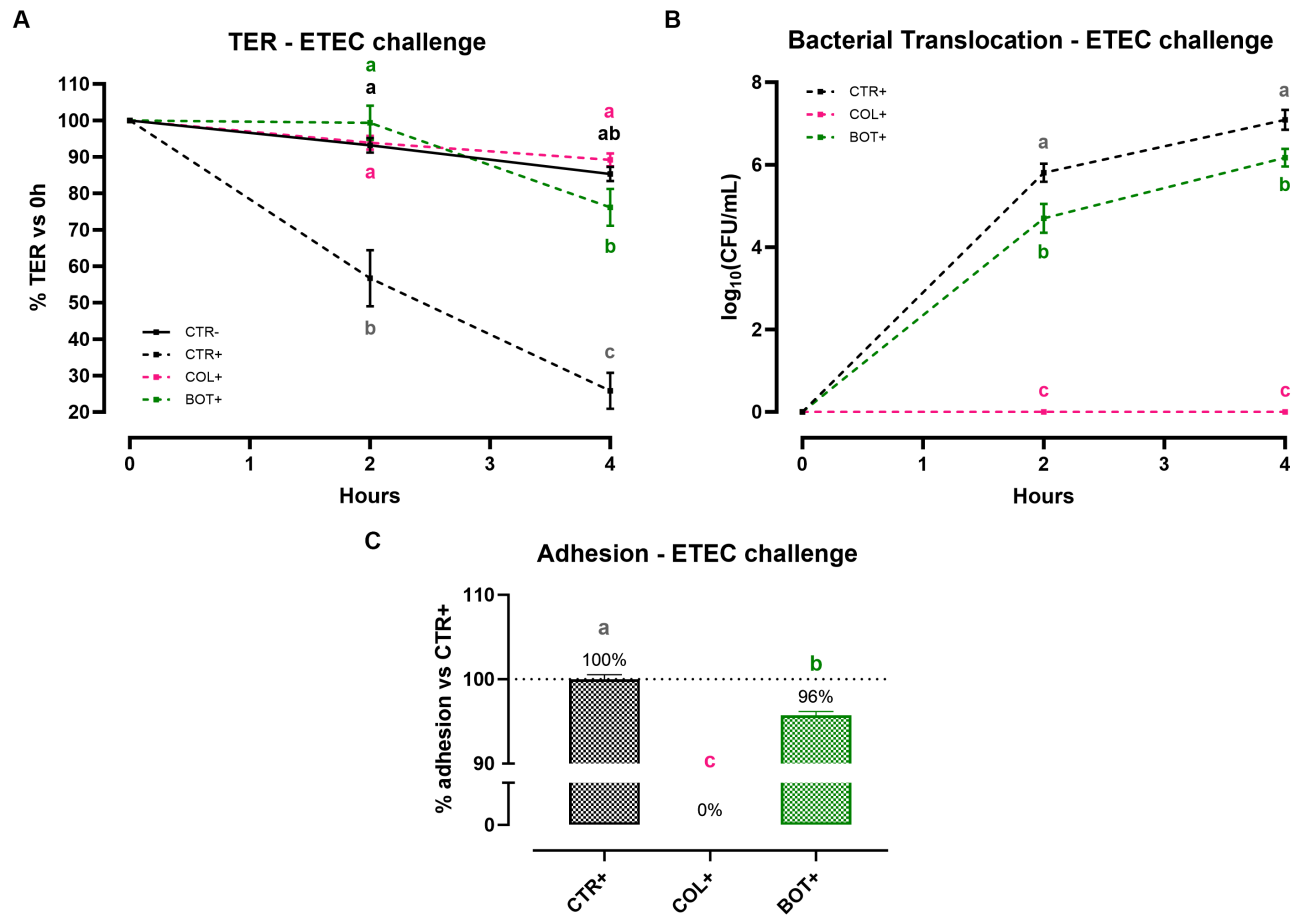
TER **(A)**, bacterial translocation **(B)**, and ETEC adhesion **(C)** assay results after an enterotoxigenic *E. coli* infection on Caco-2 cells treated with a blend of botanicals (BOT). Experimental groups with a “+” in the name were infected. Data in graphs are represented as means ± SEM. Different letters indicate significant differences with *p* < 0.05 at each timepoint.

Figure 6A reports the results of Caco-2 cellular integrity during an ETEC challenge in the presence of BOT. Data show that ETEC significantly reduced TER of the epithelial monolayer at both 2 h and 4 h after the beginning of the infection. Damages were particularly pronounced at the end of the experiment, where the TER for the CTR+ group was only at 26% of the starting value. On the contrary, the addition of BOT to the system significantly protected cellular integrity throughout the entire experiment: TER values of the BOT+ group were in line with the negative control and colistin (COL+) at 2 h, with a slight reduction in TER only registered at 4 h, but still at values statistically equal to healthy unchallenged cells (CTR-).

To investigate the ability of BOT to influence the passage of bacteria across the Caco-2 monolayer, the bacterial translocation of ETEC was also assessed, and the results are presented in Figure 6B. Data demonstrate that, while colistin – at bactericidal dose – was able to completely avoid ETEC translocation across cells, the addition of BOT to the challenge allowed a significant reduction of the viable bacteria passage across Caco-2 at both time points. At 2 h, the reduction exerted by BOT was equal to 1 log_10_(CFU/mL), which was maintained at 4 h (0.9 log_10_(CFU/mL)).

Figure 6C displays the outcomes of the adhesion assay, performed to study BOT interference in the interaction between Caco-2 cells and ETEC. The blend of botanicals significantly reduced the adhesion of the bacteria to Caco-2 enterocytes (−4% compared to control), while colistin – at a bactericidal dose – completely inhibited its attachment.

#### 3.2.2. Gene expression

**Figure 7:**
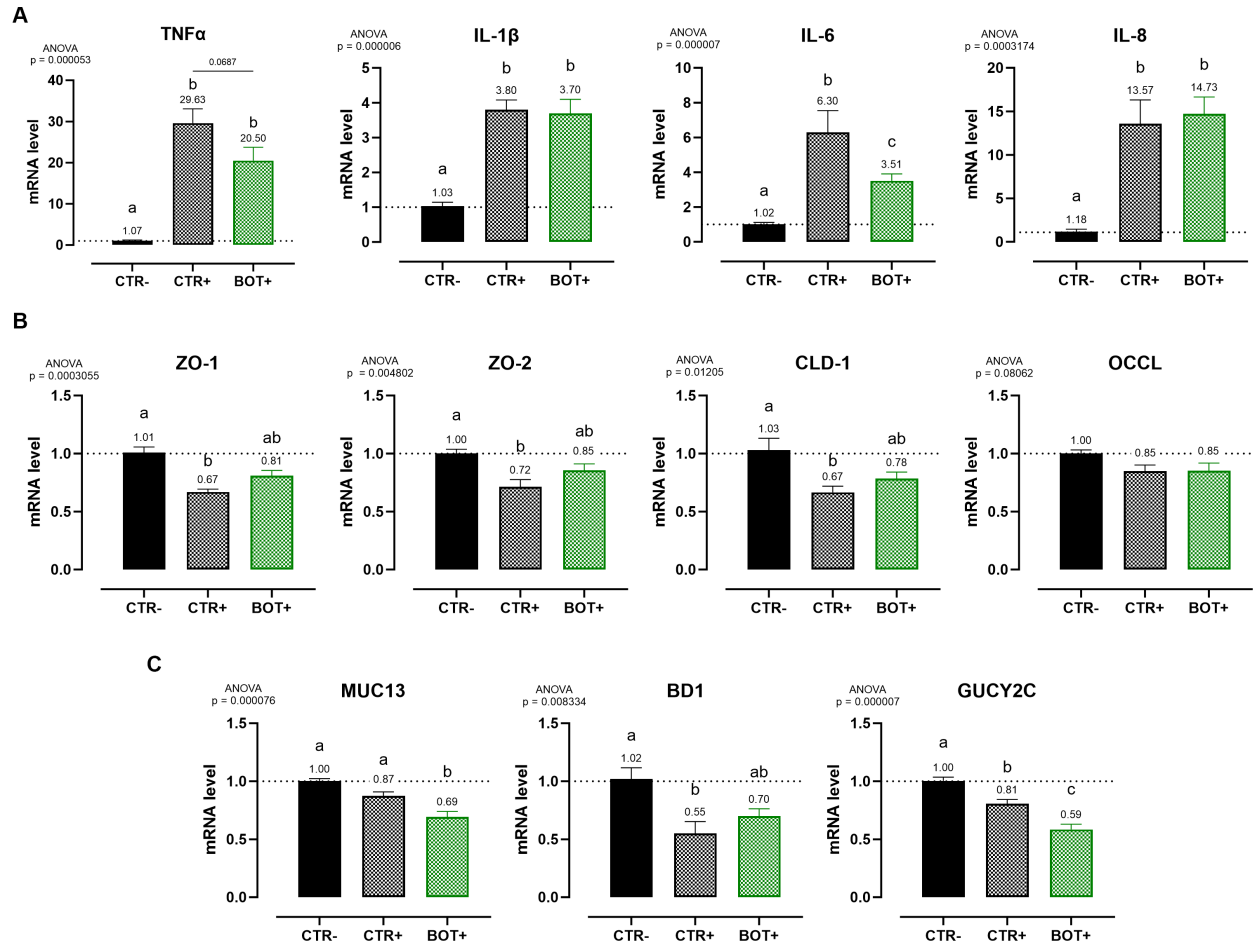
Gene expression analysis of Caco-2 cells treated with a blend of botanicals (BOT) and challenged with enterotoxigenic *E. coli* for 4 h. Panel **(A)** displays the inflammatory cytokines results (TNFα, IL-1β, IL-6, IL-8), panel **(B)** reports the data for tight-junction markers (ZO-1, ZO-2, CLD-1, OCCL), while panel C shows MUC13, BD1, and GUCY2C, three genes related to cellular response to bacterial infection. Groups with bacterial infection are represented with a “+” in the name. Data in the graphs are represented as means ± SEM. Different letters denote significant differences with *p* < 0.05; tendencies (*p* < 0.1) are highlighted by reporting their respective *p* values and indicated by a line spanning the two evaluated groups.

At the end of the bacterial challenge, cells were harvested and mRNA extracted to investigate the expression of inflammatory (Figure 7A) and tight-junction (Figure 7B) related genes. Moreover, three genes related to the cellular response to bacteria were investigated, being mucin 13 (MUC13), bacterial beta defensin 1 (BD1), and guanylate cyclase 2C (GUCY2C) (Figure 7C).

The ETEC challenge significantly increased the expression of all the analyzed pro-inflammatory cytokines when compared to negative control. The application of BOT was not able to reduce the levels of IL-1β and IL-8 expression, while it tended to decrease TNFα (*p* < 0.1) and significantly lowered (*p* < 0.05) IL-6. The blend of botanicals also improved ZO-1, ZO-2, and CLD-1 expression, originally impaired by the presence of the ETEC challenge, bringing their levels between positive (CTR+) and negative (CTR-) controls.

During the challenge, BOT reduced the expression of MUC13, a putative receptor for ETEC on enterocytes. Moreover, the GUCY2C receptor for heat-stable ETEC toxins, while being already lowered by the challenge itself, was further decreased by BOT. Finally, the expression of BD1, impaired by the bacterial challenge, was partially restored by the addition of BOT to the system.

#### 3.2.3. Immunofluorescence staining

**Figure 8:**
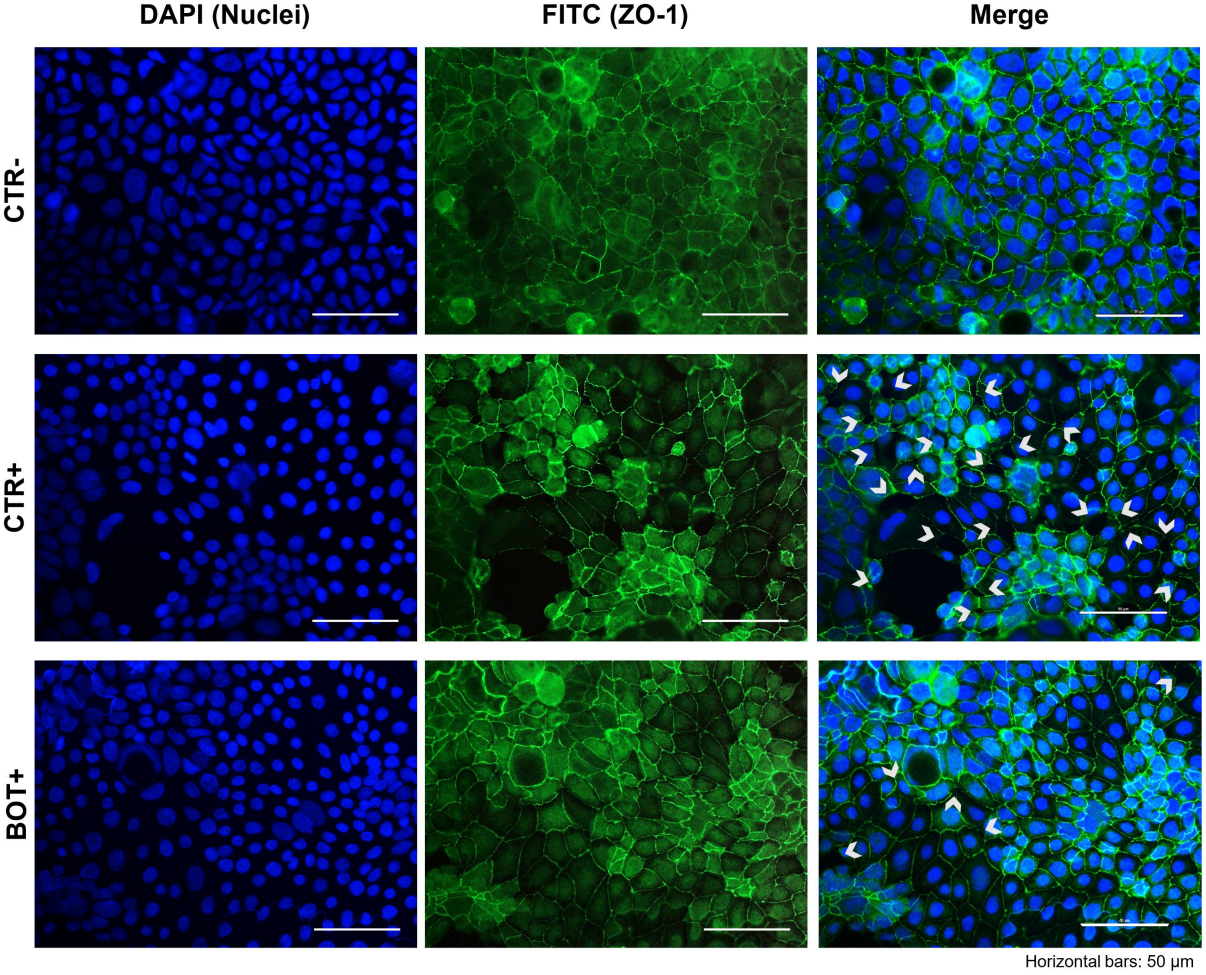
Immunofluorescence staining of Caco-2 cells treated with a blend of botanicals (BOT) and challenged with enterotoxigenic *E. coli* for 2 h. Groups with bacterial infection are represented with a “+” in the name. Left pictures show the DAPI staining of cell nuclei, the central column depicts ZO-1 staining with FITC, while the right column displays the merge of the first two images. White arrows identify areas with anomalies in ZO-1 disposition, or loss of cells. Each row displays a different experimental group.

The immunofluorescence staining results for ZO-1 of cells challenged with ETEC are shown in Figure 8. The ETEC challenge produced significant damage to the Caco-2 epithelial monolayer: compared to the healthy control, infected cells showed a swelling morphology with an irregular disposition of ZO-1 on cellular borders. Moreover, areas of cell-to-cell loss of contact left evident gaps between adjacent cell bodies. In certain areas, the degree of the damages reached a considerable extent, leading to cellular death, with acellular spaces open for bacterial translocation. The addition of BOT to the system significantly reduced the impairments exerted by ETEC, allowing the maintenance of a higher cellular tightness, with minor localized sites of ZO-1 irregular distribution or loss of contact between cellular margins. No areas of extensive cellular detachment were detected.

## 4. Discussion

Inflammation is a physiological response that occurs when the body needs to fight stressors (34, 35). In animal productions, common practices expose animals to a vast array of harmful stimuli, that can considerably impair their overall health and performance, with huge costs for producers (36, 37). In the pig productive cycle, the most delicate phase is weaning: its early occurrence elicits the onset of intestinal inflammation as a way to react to stress and adapt to the new dietary, environmental, and physiological conditions (10, 38). Caco-2 cells represent a recognized *in vitro* model to recreate a functional intestinal epithelium and to study its response against different detrimental stimuli (39, 40). The mix of cytokines and bacterial LPS employed in our investigations effectively recreated on intestinal cells the harmful state that piglets’ gut undergoes at weaning: the health status of Caco-2 enterocytes was profoundly impaired–in the acute challenge model–as demonstrated by TER, a direct indicator of epithelial tightness. However, rather than acute, it is prolonged stress that is particularly common in actual pig husbandry, since stressors usually lasts for several days and need time to be completely resolved, especially during unfavorable periods like weaning (41). For this reason, we additionally developed a chronic challenge model, that equally proved efficient in reducing epithelial integrity of Caco-2 cells throughout time, while also activating the innate immune response of enterocytes.

The high interdependence and inverse relationship between tight junctions’ state and inflammation are widely recognized in literature (42, 43). After the challenge, the drop in cellular integrity shown by our data is accompanied by the activation of a strong inflammatory status, proved by the increase of TNFα, IL-6, and IL-8 cytokine expression, and the subsequent deterioration of tight-junction mRNA levels. Consequently, the stimulation of the local inflammatory reaction triggers the release of reactive oxygen species (ROS), creating a self-amplifying cycle that further impairs homeostasis, leading to the loss of gut integrity and thus paving the way for pathogen’s colonization of the intestinal mucosa (44). Our *in vitro* inflammatory challenge model significantly increased the ROS levels in Caco-2 cells, confirming the close connection between inflammation and oxidation, with the second being a physiological outcome of the first, but at the risk of excessively stimulating the overall stress response.

Historically, the employment of pharmacological doses of zinc oxide during the weaning phase was effective in reducing diarrheal symptoms and maintaining good zootechnical parameters despite the dramatic changes piglets must face (45, 46). The mechanism of action of zinc oxide is multi-factorial, and targets several sites of the gastrointestinal tract, avoiding excessive inflammation and ROS production (16). However, the excess of zinc oxide above nutritional requirements is excreted in feces, with severe environmental and bacterial resistance concerns (16, 47, 48). Thus, the European authorities banned zinc oxide medicinal doses in 2022, while other countries are moving towards the same direction (49). Therefore, farmers are now requested to face weaning stress issues without pharmacological zinc oxide. Recognized powerful alternatives come from the bioactive compounds naturally available in fruits and plants, thanks to their diverse mechanisms of action that span both the pathogen and the host sides of the issue (19, 50, 51). When employed alone, single botanicals exert considerable supportive actions to maintain intestinal health and control bacterial overgrowth (52). Thymol, the main component of thyme essential oil, has a powerful antimicrobial and virulence-modulating action, and it is well known for its anti-inflammatory potential (53, 54). Capsaicin derived from capsicum oleoresin is a fine regulator of tight junction structure, owing also to its anti-inflammatory action and its ability to interact with the endocannabinoid system (55–57). Finally, the wide array of polyphenols inside grape seed extracts not only control bacterial toxin activity, but also exert a strong anti-inflammatory and antioxidant effect (58–60). In our study, we wanted to test the combination of the three botanicals (thymol, grape seed extract, and capsicum oleoresin; BOT) in different challenge models, complementing the individual efficacies of the single components.

The treatment with BOT was effective in protecting intestinal cells from the loss of integrity generated by an inflammatory challenge. In the case of enterocytes undergoing an acute challenge, the addition of BOT even allowed a faster recovery to normal integrity values. This beneficial effect was confirmed also when Caco-2 was pre-treated with the blend of botanicals: the pre-treatment with BOT improved the integrity of healthy cells before the challenge, and helped enterocytes to better deal with the inflammatory stimulus. In both acute challenges, even if still present, the drop in TER in BOT-treated groups was significantly limited.

When the inflammation was prolonged over time to recreate a chronic challenge model, BOT consistently supported enterocytes’ health across the entire experiment, allowing cells to maintain a higher degree of tightness, and stimulating a complete recovery of TER if given sufficient time. Since weaning inflammation in pigs usually lasts for several days and requires time to be resolved (6, 61), the BOT-iterated supportive action is of central interest to maintain a healthier state, a higher animal performance, and to accelerate the recovery process to a homeostatic condition. The beneficial effects of BOT are related to its wide action against enterocytes’ inflammatory response. Gene expression data at the end of the chronic challenge demonstrate that BOT significantly reduced the expression of pro-inflammatory cytokines like IL-6 and IL-8, while also numerically decreasing TNFα and IL-1β. These results are likely a consequence of thymol and capsaicin action, two BOT ingredients. The two terpenes possess phenolic hydroxyl groups that inhibit kinases responsible for NF-kB activation, one of the key transcription factors that elicit stress responses in cells (62, 63). Moreover, thymol and capsaicin directly interfere with NF-kB active sites, avoiding its translocation in the cell nucleus and the consequent inflammatory stimulation (64, 65). Finally, the two bioactives modulate the endocannabinoid system by binding TRPV1 and 3 receptors, triggering their anti-inflammatory potential (66–68). The lowered inflammatory tone enabled the preservation of several tight junction components: during the challenge, BOT maintained higher expression levels of ZO-1 and-2, two scaffold-like proteins, and CLD-1 and OCCL, two sealing elements (69). These figures were also confirmed by the immunofluorescence staining for ZO-1: BOT-treated challenged cells showed a better tight junction distribution and only minor areas of loss of contact between adjacent cells. Since cytokines impair the distribution and function of tight junctions (70), the reduced inflammatory degree by BOT resulted in the preservation of a higher epithelial integrity and structure.

During the inflammatory cascade, the triggering of NF-kB stimulates the synthesis of oxidative enzymes to produce ROS as a physiological component of the inflammatory response (71, 72). However, the persistent accumulation of ROS promotes the activation of the inflammasome, with the onset of a loop that further exacerbates inflammation (73, 74). Polyphenols contained inside BOT may contribute to the disruption of this self-amplifying cycle (60, 75, 76). Our data showed that BOT reduced ROS generated by the LPS and cytokine challenge: enterocytes were safeguarded from an excessive inflammatory activation, thus avoiding the subsequent ROS synthesis. Moreover, BOT decreased the oxidative stress also during an H_2_O_2_ challenge, displaying its direct detoxifying action against ROS, the molecules that fuel the pro-oxidative loop.

During inflammation, the structure and function of the intestinal mucosa is deeply impaired. When the intestinal homeostasis is lost, pathogens can take over (77, 78). Weaning stress and inflammation in piglets represent the perfect condition for the overgrowth of enterotoxigenic *Escherichia coli* (ETEC) species, whose main representative is ETEC F4^+^ (79). After adhering to the apical side of enterocytes, ETEC F4^+^ produce heat-labile and heat-stable toxins, that elicit the release of ions in the intestinal lumen and, consequently, water (11). Piglets affected by this pathogen rapidly develop post-weaning diarrhea, that markedly reduces performance parameters and increases mortality (13). The botanical-based blend used in this study was effective in protecting Caco-2 enterocytes against an *in vitro* challenge with ETEC F4^+^. As shown by TER levels, intestinal integrity of infected enterocytes treated with BOT was comparable to unchallenged cells and to cells treated with an effective antibiotic – colistin – used at a bactericidal dose. The protection of epithelial morphology was confirmed by the immunofluorescence staining of BOT treated cells, which displayed less damages and the preservation of ZO-1 disposition if compared to the infected ones. As an indirect measurement of integrity, also bacterial translocation across Caco-2 was significantly reduced by BOT, highlighting the protective action of the blend and its ability to keep a tighter cellular monolayer. Even against the pathogen infection, the precise mechanism of action of BOT appears to be directed towards the modulation of the epithelial inflammatory response: the reduced expression of TNFα and IL-6 are indicators of a decreased inflammatory tone. On the opposite, the maintenance of higher levels of IL-8, a chemokine that attracts other immune cells in the site of inflammation (80), and the increased amount of BD1, a defensin involved in the protection against bacterial infections (81), might be a sign of an immune boosting effect, allowing the intestinal mucosa to be ready to efficiently respond in case of an excessive bacterial infection. The ability of BOT to lower inflammation is demonstrated also by the preservation of a higher tight-junction expression, similarly to the results of the inflammatory challenge. This BOT reinforcing action on intestinal mucosa integrity counteracts the disruptive effects that ETEC pathogens have on tight junctions (82, 83).

In addition, botanicals seemed to exert an effect on the susceptibility of intestinal cells to ETEC infection, as displayed by the BOT-driven reduction of MUC 13 expression, one of the candidate receptors that ETEC F4^+^ exploits to target enterocytes (84, 85). This effect not only limits the adhesive capacity of the pathogen to Caco-2 cells, but opposes also NF-kB activity, mitigating the intestinal cell inflammatory response (86). This outcome further complements the available evidence about the virulence-modulating activity of the components inside BOT: thymol, polyphenols, and capsaicin interfere with the expression and activity of ETEC F4^+^ toxins and adhesins (22, 24, 87, 88). Even if the bioactives inside BOT were used at sub-inhibitory doses, not directly able to prevent bacterial growth, it is likely possible that those low concentrations were still capable of decreasing ETEC ability to generate damages on the enterocytes, thus limiting the intestinal inflammatory response and the activation of ion channels responsible for the onset of diarrhea *in vivo*. Moreover, the BOT beneficial effect also extends to other markers, like GUCY2C, the receptor for the heat-stable STa bacterial toxin: data showed a significant decrease in its expression during the challenge, with a trend particularly marked in the BOT group. GUCY2C reduced signaling is physiologically linked to the stimulation of epithelial growth and regeneration: its downregulation might be correlated to an improved healing of damaged sites on intestinal surface, with the overall maintenance of an enhanced mucosal integrity (89, 90). Therefore, in the context of a bacterial infection, BOT displays interesting effects on both the host and the pathogen side, deeply modulating their interaction at multiple levels.

## 5. Conclusion

In conclusion, the blend of botanicals (BOT) was effective in controlling acute and chronic stress originating from inflammatory and ETEC bacterial challenges, that strongly affect pigs at weaning. The mechanism of action of BOT combines the individual features of its single components to preserve epithelial integrity. In particular, BOT modulated enterocytes’ inflammatory response, protected tight junction expression and function, controlled cellular oxidative stress, and reduced epithelial susceptibility against pathogens, while simultaneously acting on bacteria adhesive capacity. These outcomes enlighten the multifaceted activity of BOT at different stages of the host-pathogen interaction and support its employment in the feed to manage post-weaning diarrhea and weaning stress in piglets. Further studies should now explore BOT efficacy *in vivo*, both in field trials and challenge models. Moreover, other *in vitro* research could be addressed towards the investigation of BOT beneficial effects on additional complex models like intestinal organoids, and other cell lines like immune or liver cells, considering their role in the greater host response against stress.

## Data availability statement

The raw data supporting the conclusions of this article will be made available by the authors, without undue reservation.

## Ethics statement

Ethical approval was not required for the studies on humans in accordance with the local legislation and institutional requirements because only commercially available established cell lines were used.

## Author contributions

AB: Conceptualization, Investigation, Writing – original draft, Methodology. AT: Conceptualization, Investigation, Writing – original draft. BT: Writing – review & editing. AP: Supervision, Writing – review & editing. EG: Conceptualization, Supervision, Writing – review & editing.
